# High Interleukin (IL)-6 is Associated with Lower Lung Function and Increased Likelihood of Metabolic Dysfunction in Asthma

**DOI:** 10.1007/s41030-024-00281-z

**Published:** 2024-12-23

**Authors:** Dionne Adair, AmirBehzad Bagheri, Matheos Yosef, Shokoufeh Khalatbari, Toby Lewis, Arjun Mohan, Njira Lugogo

**Affiliations:** 1 Division of Pediatric Pulmonary, Medical College of Georgia, Augusta, GA USA; 2 Division of Pulmonary and Critical Care Medicine, University of Michigan, Ann Arbor, MI USA; 3 Michigan Institute for Clinical and Health Research, University of Michigan, Ann Arbor, MI USA; 4 Division of Pediatric Pulmonology, University of Michigan, Ann Arbor, MI USA

**Keywords:** Asthma, Interleukin-6 (IL-6), Obesity, Inflammation, Metabolic dysfunction

## Abstract

**Introduction:**

Asthma is a complex condition characterized by airway inflammation. Interleukin-6 (IL-6) plays a significant role in asthma pathogenesis through its effects on T cells and its association with pro-inflammatory responses. Both lung and circulating IL-6 levels are elevated in asthma. IL-6 is positively associated with disease severity, frequent exacerbations, and impaired lung function, all of which can be observed clinically. We developed an IL-6 cut-off model to examine the association between high IL-6, race, high body mass index (BMI), metabolic disease, and asthma severity as assessed by reduced lung function.

**Methods:**

This study utilized the Coronary Artery Risk Development in Young Adults (CARDIA) database, comprised of 5115 adults, to investigate the relationship between IL-6 levels, asthma, race, and metabolic dysfunction. A "healthy" subset of 427 patients was used to compute the IL-6 cut-off. IL-6 levels within detection limits (0.15–12 pg/mL) were analyzed. The IL-6 cut-off was determined using the 95th percentile of log-transformed IL-6 values for lean (BMI < 25) and healthy individuals. Specific cut-offs were established for racial groups. Statistical analyses involved comparing patient characteristics between high and low IL-6 groups, regression analyses, and assessment of factors influencing lung function changes.

**Results:**

Using an IL-6 cut-off of 4.979 pg/mL, the cohort was divided into high and low IL-6 groups. High IL-6 correlated with Black race, higher BMI, hypertension, and markers of metabolic dysfunction, e.g., elevated HbA1c, C-reactive protein (CRP), and reduced lung function. Multivariable analysis linked high IL-6 with male gender, high BMI, Black race, HbA1c, CRP, and inversely with lung function and total cholesterol. Obesity showed a consistent positive association with elevated IL-6, regardless of the presence or absence of asthma. Patients with asthma and high IL-6 were more likely to be Black and showed increased CRP. Lung function was lowest in non-lean, high IL-6 patients with asthma, with similar trends in non-lean (BMI ≥ 25) patients without asthma.

**Conclusion:**

This study underscores the significant association between IL-6, asthma, obesity, and metabolic dysfunction. Elevated IL-6 correlates with asthma severity, particularly in individuals with obesity. Future research should explore anti-IL-6 therapies for specific phenotypes, such as obesity-related asthma. These findings advance our understanding of asthma and the role of IL-6 in its pathogenesis.

**Supplementary Information:**

The online version contains supplementary material available at 10.1007/s41030-024-00281-z.

## Key Summary Points

**Table:** 

***Why carry out this study?***
Our understanding of the role of IL-6 (interleukin-6) in asthma is evolving, and this longitudinal study was an ideal way to determine the association between IL-6 and important asthma outcomes such as lung function.
Our hypothesis is that high IL-6 levels are associated with increased metabolic dysfunction and poorer asthma outcomes, particularly in higher-risk populations such as those with obesity.
***What was learned from the study?***
Our study revealed that elevated IL-6 levels are associated with certain demographic risk factors, but also with markers of metabolic dysfunction and inflammation.
Non-lean (BMI ≥ 25) patients with asthma and high IL-6 levels have the lowest lung function, indicating the presence of a more severe asthma phenotype.

## Introduction

Asthma is a common condition characterized by chronic airway inflammation that is mediated by a wide variety of cells and cytokines. In some patients, airway inflammation is primarily driven by type 2 (T2) helper cells [1] and in others by non-T2 mechanisms [2]. A subset of patients with asthma are obese, with difficult to treat symptoms and poor response to traditional treatments [3]. Interleukin-6 (IL-6) promotes differentiation of T helper cells into pro-inflammatory subtypes, inhibits interferon (IFN)-γ signaling, and inhibits generation of regulatory T cells (Treg) [4–7]. IL-6 has increased expression in individuals with obesity [8, 9]. Several observations also suggest that IL-6 may play a key role in asthma pathogenesis, particularly in patients with obesity [8–12]. Lung and circulating IL-6 levels are also up-regulated in other asthma phenotypes, for example, in non-allergic asthma and patients with predominantly neutrophilic or mixed granulocytic inflammation [10–12]. Clinically, increased IL-6 levels are associated with severe disease with more frequent exacerbations, lower lung function, and poorer asthma control [10, 13].

There is increasing prevalence of metabolic dysfunction in parallel with the increasing rates of obesity [14, 15]. Patients with obesity, asthma, and metabolic dysfunction have more severe asthma, compared to non-obese patients with asthma but without metabolic dysfunction [13]. The Black population is disproportionately affected by obesity, metabolic dysfunction, and asthma [16]. Finally, there is some suggestion of a racial impact on IL-6 levels, with the Black population having higher levels than their white counterparts [14]. In order to explore the associations between IL-6, asthma, and metabolic dysfunction, we utilized the Coronary Artery Risk Development in Young Adults (CARDIA) study database https://www.cardia.dopm.uab.edu [17]. We devised a model to obtain a cut-off for IL-6 and compared high and low IL-6 groups.

We hypothesized that high IL-6 levels would be associated with Black race, high body mass index (BMI), and metabolic dysfunction. We also aimed to show that high IL-6 is associated with asthma severity, as assessed by lung function measures.

## Methods

### Study Population

The CARDIA database is a longitudinal study of 5115 non-Hispanic Black and non-Hispanic white adult men and women recruited in 1985–1986 at the age of 18–30 years old, and followed to examine the development and determinants of clinical and subclinical cardiovascular disease (CVD). In this cohort, patient history of asthma and some asthma related data were collected, as was lung function, markers of inflammation, i.e., C-reactive protein (CRP), serum IL-6, and indicators of metabolic syndrome/dysfunction, including a history of diabetes, hypertension, and hypercholesterolemia.

Our analysis was carried out on Year 20 data, as this was the time point at which IL-6 levels were tested. Demographic (age, sex, race) data, cardiovascular risk factors, self-reported chronic co-morbidities including asthma, hypertension and diabetes, lung function, and markers of inflammation were collected at this time point. These data also included responses from asthma related questions including age of diagnosis, symptoms in the past year, and medication usage.

**Fig. 1:**
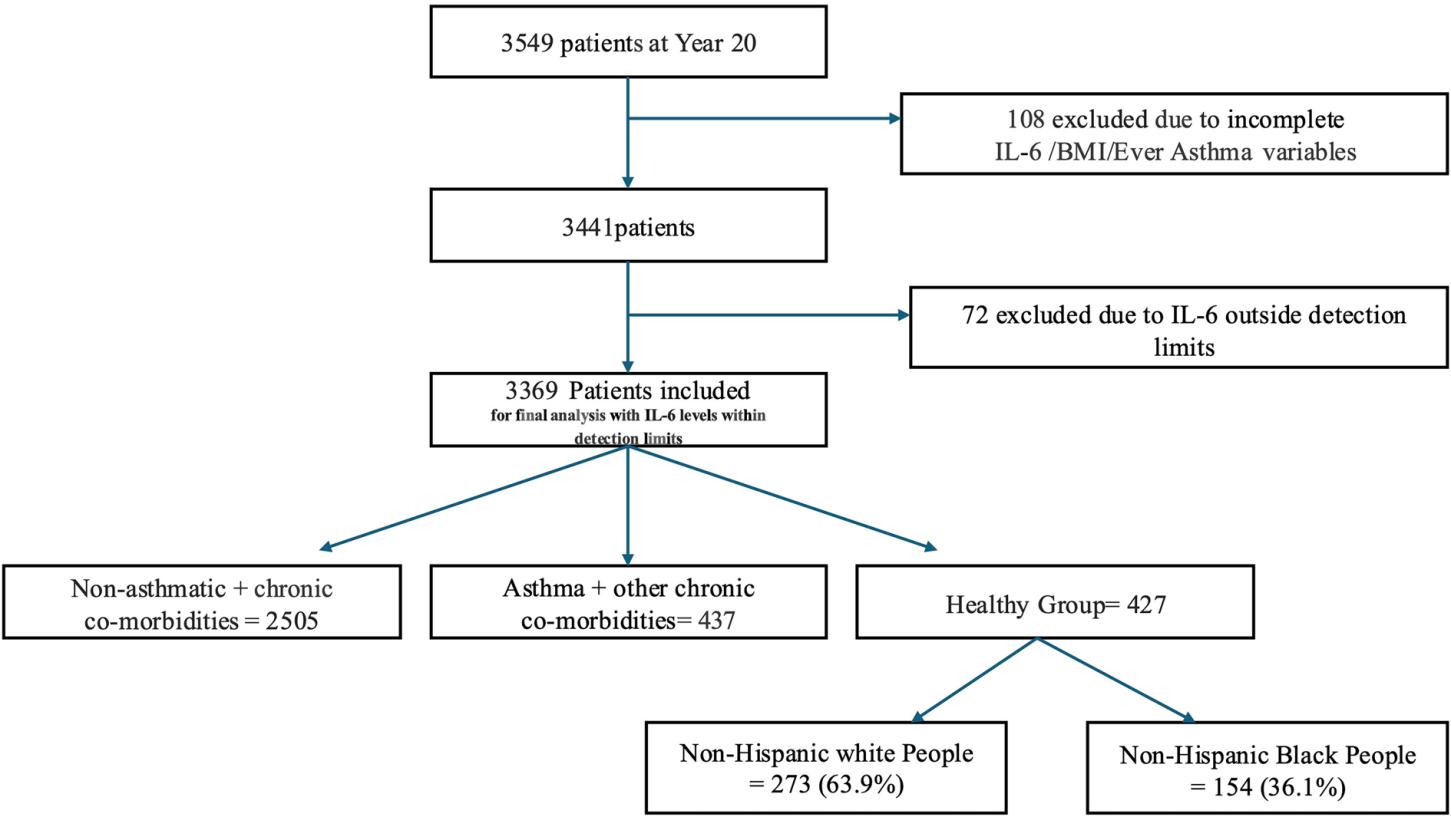
Schematic of patients included in the final analysis; *IL-6* interleukin 6, *BMI* body mass index

Of the original 5115 patients at the start of the study, 3549 patients remained in the study at Year 20. We excluded 180 patients who were missing asthma diagnoses, BMI, or IL-6 levels, or whose IL-6 levels were outside detection limits of 0.15–12, resulting in a cohort of 3369 patients (Fig. 1).

### Study Design

We identified a “healthy” subset of 427 patients (154 Black, 273 white) from the database to calculate our IL-6 cut-off. Healthy patients were defined as lean (BMI < 25), patients without asthma who had neither metabolic dysfunction nor any other chronic medical condition. For a complete list of excluded chronic conditions, see Supplement (Table S1). The entire cohort of patients (*n* = 3369), with IL-6 levels within detection limits was then classified as high versus low IL-6 groups using the calculated cut-off to determine the relationship between IL-6, patient characteristics, asthma outcomes, and metabolic dysfunction.

### Computation of IL-6 Cut-off

To determine the IL-6 cut-off used to divide the population into high versus low IL-6 groups, we followed the guidelines of the national committee for clinical laboratory standards (IL-6 levels for the CARDIA cohort were determined using the R&D Systems HS600B HS Elisa assay). The detection range for the assay was 0.15–12 pg/mL. Data extrapolation beyond that range was allowed up to approximately 39 and down to approximately 0.05. This resulted in IL-6 concentrations between 0.4 and 39.842 pg/mL. For the purposes of our study and IL-6 cut-off determination, we included those within detection limits. Despite these cut-offs, we captured 98% of patients. IL-6 levels were first log-transformed to normalize their distribution. The 95th percentile of the log IL-6 values (within the detection limit of the instrument used) was computed for individuals in the cohort who were lean (BMI < 25) and healthy (as defined above), and then back-transformed to obtain the IL-6 cut-off (directly computing the 95% of the IL-6 values also gave the same result). For a comparison analysis, race-specific cut-offs for IL-6 were computed for the non-Hispanic Black and non-Hispanic white groups (high IL-6: IL-6 > 7.232 pg/mL for Black people, IL-6 > 3.453 pg/mL for white people; IL-6 > 4.979 pg/mL overall).

### Statistical Analysis

All statistical analyses were conducted on data restricted to IL-6 within the detection limits (0.15–12 pg/mL) of the instrument (R&D Systems HS600B HS Elisa) used for measurement. Patient demographic and clinical characteristics (Table 1) were summarized as counts and percentages for categorical data and median and interquartile range (IQR) for continuous data, and compared between high and low IL-6 groups using chi-squared or Fisher’s exact tests for categorical and Wilcoxon rank sum tests for continuous data. Cross-sectional (at Year 20) effects of patient characteristics on IL-6 levels were assessed using univariable and stepwise multivariable ordinary (continuous IL-6 data) and logistic regression (high vs. low IL-6) analyses using backward elimination. The above univariable and multivariable analyses were repeated on the subset of the asthma population. The following characteristics were included as predictors: sex, age (at Year 20), race (Black vs. white), BMI, metabolic dysfunction, CRP, forced expiratory volume in one second (FEV_1_), forced vital capacity (FVC) as well as FEV_1_/FVC ratio, hemoglobin A1C (HbA1c), asthma diagnosis, current asthma (obtained from two characteristics: asthma in past 1 year and whether the patient is currently taking medication for asthma), systolic blood pressure (SBP), diastolic blood pressure (DBP), metabolic dysfunction, hypertension, and cholesterol. Univariable and multivariable analyses were also conducted on lung function parameters to determine factors strongly associated with changes in FEV_1_ and FVC as outcomes.

**Table 1:** Patient characteristics by IL-6 level (at Year 20)

Characteristic	High IL-6^a^ (*n* = 305)	Low IL-6 (*n* = 3064)	Total (*n* = 3369)	*p* value
Age at Year 20 (years) (median, IQR)	45 [43–49]	46 [42–48]	46 [42–48]	0.664
Sex (%)				0.001
Female	199 (65.2%)	1703 (55.6%)	1902 (56.5%)	
Male	106 (34.8%)	1361 (44.4%)	1467 (43.5%)	
Race (%)				< .001
Non-Hispanic Black	203 (66.6%)	1341 (43.8%)	1544 (45.8%)	
Non-Hispanic white	102 (33.4%)	1723 (56.2%)	1825 (54.2%)	
BMI (kg/m^2^) (median, IQR)	31.85 [26.90–40.25]	27.82 [24.29–32.23]	28.08 [24.51–32.82]	< .001
Asthma	47 (15.4%)	390 (12.7%)	437 (13.0%)	0.184
Asthma in past year	29 (9.6%)	204 (6.7%)	233 (7.0%)	0.058
Currently taking meds for Asthma^b^	32 (10.5%)	197 (6.4%)	229 (6.8%)	0.007
2 or more wheezing attacks	38 (12.8%)	297 (9.8%)	335 (10.1%)	0.101
HTN^b^ (SBP ≥ 130 and DBP ≥ 80)	51 (16.8%)	419 (13.7%)	470 (14.0%)	0.138
High blood pressure (Y/N)	111 (36.9%)	680 (22.4%)	791 (23.7%)	< .001
HbA1c (%) (median, IQR)	5.6 [5.3–6]	5.3 [5.1–5.6]	5.4 [5.1–5.7]	< .001
Fasting glucose (mg/dL) or (mmol/dL) (median, IQR)	95 [88–105] or 5.27 [4.88–5.83]	93 [87–100] or 5.16 [4.83–5.55]	94 [87–101] or 5.22 [4.83–5.61]	0.022
Cholesterol levels mg/dL and mmol/L (high cholesterol %)^b^	52 or 1.35 (17.6%)	705 or 18.26 (23.9%)	757 or 19.61 (23.3%)	0.016
Total plasma cholesterol (mg/dL) or (mmol/dL) (median, IQR)	177 [155–203] or 4.58 [4.01–5.26]	184 [163–208] or 4.77 [4.22–5.39]	184 [162–208] or 4.77 [4.2–5.39]	0.001
Average SBP (mm HG) (median, IQR)	117 [107–126]	115 [106–124]	115 [106–124]	0.029
Average DBP (mm HG) (median, IQR)	75 [67–84]	72 [65–80]	72 [65–80]	< .001
Metabolic dysfunction^b^ (Y/N) (%)	95 (31.1%)	574 (18.7%)	669 (19.9%)	< .001
CRP (ug/mL) (median, IQR)	4.29 [1.42–9.59]	1.02 [0.44–2.55]	1.12 [0.48–3.02]	< .001
FEV_1_ (L) (median, IQR)	2.59 [2.20–3.17]	2.99 [2.52–3.60]	2.96 [2.48–3.57]	< .001
FVC (L) (median, IQR)	3.31 [2.75–4.10]	3.83 [3.19–4.61]	3.78 [3.14–4.56]	< .001
FEV/FVC (median, IQR)	0.80 [0.75–0.84]	0.79 [0.75–0.83]	0.79 [0.75–0.83]	0.737
Current asthma (%)	38 (12.5%)	249 (8.1%)	287 (8.5%)	0.010

All ‘Not sure’ values were replaced by missing so all percentages (%) are out of total non-missing values. The entries are *n* (%) with chi-square *p* values for categorical characteristics and median (IQR) with Wilcoxon two-sample test *p* values for continuous characteristics

*BMI* body mass index, *CRP* C-reactive protein, *DBP* diastolic blood pressure, *FEV* forced expiratory volume, *FEV1* forced expiratory volume in 1 s, *FVC* forced vital capacity, *HbA1c* glycated hemoglobin, *HTN* hypertension, *SBP* systolic blood pressure

^a^High IL-6 is defined as IL-6 > 4.979 pg/mL, otherwise low IL-6

^b^Metabolic syndrome defined as 3 or more of the following: abdominal obesity; hypertriglyceridemia; HDL < 40 or < 50 mg/dL (female and male, respectively); BP ≥ 130/85; fasting glucose of 100 mg/dL or greater

Univariable and stepwise multivariable analyses were conducted on all the available relevant characteristics for each outcome mentioned above. A *p* value less than 0.05 was considered statistically significant for all hypothesis tests. No adjustments were made for multiple hypotheses testing. Analyses were performed using SAS^®^ version 9.4 (Cary, NC, USA).

### Ethical Approval

The data used were non-identifiable and the database is publicly available.

## Results

Using the calculated IL-6 cut-off value (4.979 pg/mL), the cohort was divided into high (305/3369) and low IL-6 groups (3064/3369) (Table 1). High IL-6 patients did not differ from low IL-6 patients in age, asthma diagnosis, or percentage of patients reporting asthma symptoms in the past year. However, the high IL-6 group was more likely to be Black (66.6% vs. 43.8%, *p* < 0.001), have higher BMI [median 31.85 (26.90–40.25) kg/m^2^ vs. 27.82 (24.29–32.23) kg/m^2^, *p* < 0.001], have high blood pressure (36.9% vs. 22.4%, *p* < 0.001), higher rates of metabolic dysfunction (31.1% vs. 18.7%, *p* < 0.001), higher HbA1c [5.6 (5.3–6) vs. 5.3 (5.1–5.6), *p* < 0.001], higher CRP (4.29 (1.42–9.59] vs. 1.02 (0.44–2.55], *p* < 0.001) and lower lung function as evidenced by lower FEV_1_ [2.59 (2.20–3.17) vs. 2.99 (2.52–3.60), *p* < 0.001] and FVC [3.31 (2.75–4.10) vs. 3.83 (3.19–4.61), *p* < 0.001] (Table 1).

**Fig. 2:**
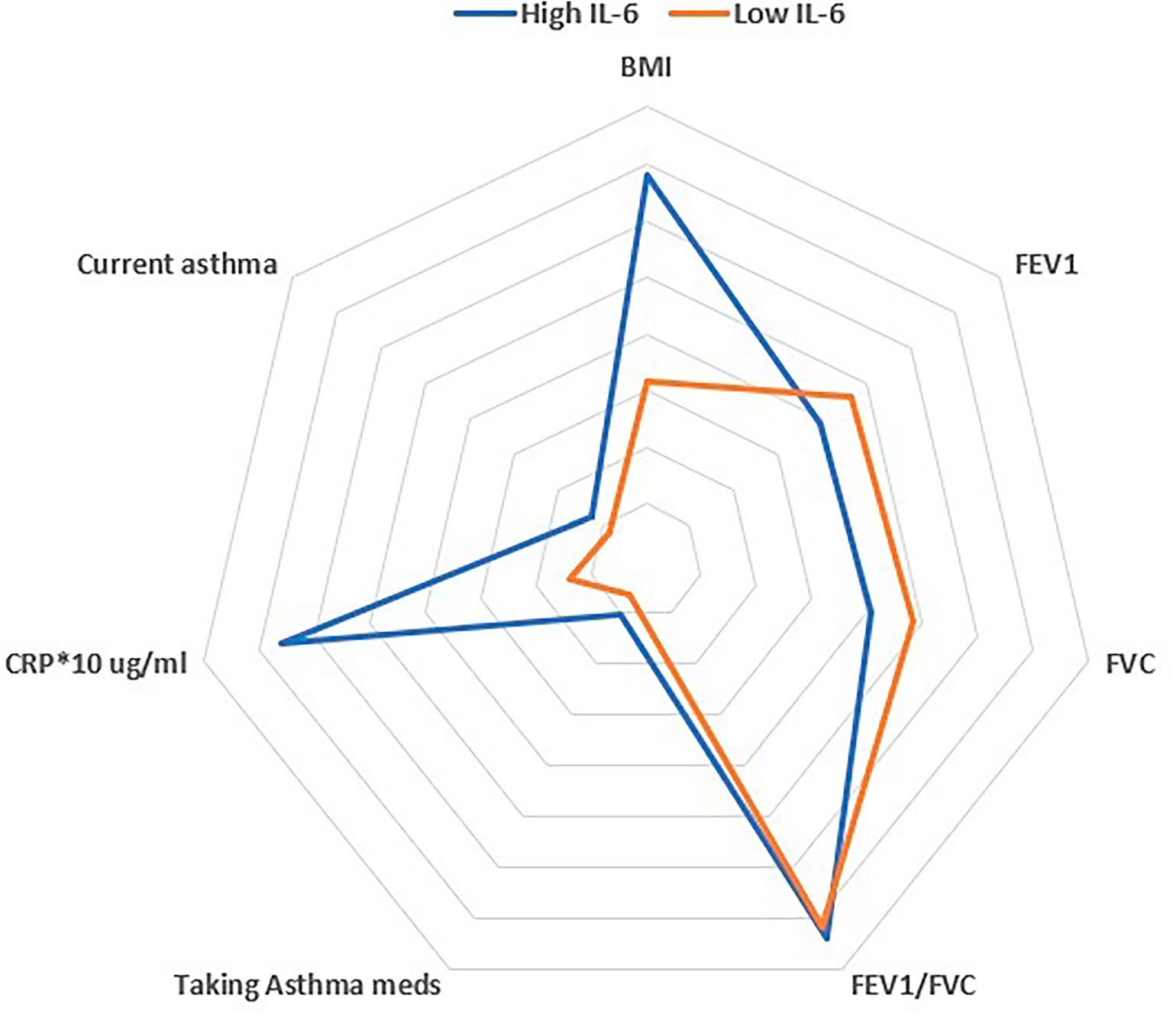
Spider plot showing factors associated with high and low IL-6 in our multivariate model. Variables are normalized based on minimum and maximum of the variables between 0 and 10. *CRP has been multiplied by 10 to show its clinically significant difference on the figure; *BMI* body mass index, *CRP* C-reactive protein, *FEV1* forced expiratory volume in 1 s, *FVC* forced vital capacity, *IL-6* interleukin 6

Multiple factors were significantly associated with high IL-6, in the univariable logistic models, including race, sex, metabolic dysfunction, and active asthma (Table 2). However, in the multivariable model male sex (OR 1.473; 95% CI 1.02–2.128), higher BMI (OR 1.024, 95% CI 1.002–1.04), Black race (OR 1.413, 95% CI 1.030–1.939), HbA1c (OR 1.302, 95% CI 1.043–1.625), and CRP (OR 1.148, 95% CI 1.116–1.182) were positively associated with the odds of high IL-6, while lung function (FEV_1_) (OR 0.693, 95% CI 0.535–0.897), total cholesterol (OR 0.993, 95% CI 0.989–0.997), and fasting glucose (OR 0.991, 95% CI 0.984–0.999) were negatively associated with the odds of high IL-6 (Fig. 2; Table 2). Given the negative association between IL-6 and lung function and the observation that Black patients generally have a higher IL-6, we investigated the possibility of an interaction effect of IL-6 and race on FEV_1_ using a multivariable regression model. The interactions were not significant.

**Table 2:** Univariable and multivariable analysis demonstrating factors associated with the odds of high IL-6 in the entire cohort

Effect	Univariable (*n* = 3369)			Multivariable (*n* = 2863)	
	*N* used	OR [95% CI]	*p* value	OR [95% CI]	*p* value
Age at Year 20 (years)	3369	1.003 [0.970, 1.036]	0.876		
Sex (female vs. male)	3369	1.500 [1.173, 1.919]	0.001	0.679 [0.470, 0.980]	0.039
Race (non-Hispanic Black vs. non-Hispanic white)	3369	2.557 [1.995, 3.278]	< .001	1.413 [1.030, 1.939]	0.032
BMI (kg/m^2^)	3369	1.097 [1.079, 1.115]	< .001	1.024 [1.002, 1.047]	0.032
2 or more wheezing attacks (yes vs. no)	3329	1.352 [0.942, 1.939]	0.102		
HTN (SBP ≥ 130 and DBP ≥ 80)	3366	1.272 [0.925, 1.748]	0.138		
HbA1C (%)	2961	1.273 [1.152, 1.406]	< .001	1.302 [1.043, 1.625]	0.019
Fasting glucose (mg/dL)	3365	1.004 [1.000, 1.007]	0.044	0.991 [0.984, 0.999]	0.033
Total plasma cholesterol (mg/dL)	3367	0.995 [0.992, 0.999]	0.010	0.993 [0.989, 0.997]	0.001
Average SBP (mm HG)	3366	1.010 [1.002, 1.017]	0.011		
Average DBP (mm HG)	3366	1.024 [1.014, 1.034]	< .001		
Metabolic dysfunction (Y/N)	3369	1.962 [1.515, 2.542]	< .001		
CRP (ug/mL)	3365	1.197 [1.169, 1.227]	< .001	1.148 [1.116, 1.182]	< .001
FEV1 (L)	3271	0.497 [0.418, 0.590]	< .001	0.693 [0.535, 0.897]	0.005
FEV/FVC	3271	1.047 [0.174, 6.289]	0.960		
Current asthma (yes vs. no)	3364	1.606 [1.116, 2.311]	0.011		

*BMI* body mass index, *CRP* C-reactive protein, *DBP* diastolic blood pressure, *FEV* forced expiratory volume, *FEV1* forced expiratory volume in 1 s, *FVC* forced vital capacity, *HbA1c* glycated hemoglobin, *HTN* hypertension, *SBP* systolic blood pressure

Separate models were also analyzed (adjusted for other significant covariates) to determine the impact of obesity and asthma on IL-6 levels. These models demonstrated that being overweight or obese is significantly associated with higher log (IL-6), regardless of the presence or absence of co-morbid asthma. In the multivariable regression model of (log)IL-6, higher BMI, Black race, increased CRP, and metabolic dysfunction were positively associated with (log)IL-6, while lung function (FEV_1_) and total cholesterol were negatively associated with (log)IL-6 (Supplement, Table S2). Multivariable analysis of FEV_1_ (Supplement, Table S3a) and FVC (Supplement, Table 3b) revealed negative associations with Black race, female sex, CRP, IL-6, HbA1c, metabolic dysfunction, age, and current asthma, and a positive association with height. FVC was additionally negatively associated with diastolic blood pressure and FEV_1_ was further negatively associated with ≥ 2 wheezing attacks (Supplement, Table 3b).

**Table 3:** Univariable and multivariable analysis demonstrating factors associated with odds of high IL-6 for patients with asthma

Effect	Univariable OR [95% CI], *p* value	Multivariable OR [95% CI], *p* value (*n* = 436)
Age at Year 20 (years)	1.066 [0.973, 1.167], 0.168	
Sex (female vs. male)	3.417 [1.414, 8.255], 0.006	
Race (non-Hispanic Black vs. non-Hispanic)	4.399 [2.071, 9.343], < .001	3.392 [1.521, 7.566], 0.003
BMI (kg/m^2^)	1.097 [1.056, 1.140], < .001	
2 or more wheezing attacks (yes vs. no)	1.303 [0.674, 2.521], 0.431	
HTN (SBP ≥ 130 and DBP ≥ 80)	1.174 [0.522, 2.640], 0.698	
HbA1C (%)	1.228 [0.985, 1.531], 0.068	
Fasting glucose (mg/dL)	1.005 [0.999, 1.012], 0.128	
Total plasma cholesterol (mg/dL)	1.000 [0.991, 1.008], 0.914	
Average SBP (mm HG)	1.015 [0.999, 1.032], 0.063	
Average DBP (mm HG)	1.029 [1.005, 1.053], 0.019	
Metabolic dysfunction (Y/N)	1.842 [0.938, 3.618], 0.076	
CRP (ug/mL)	1.225 [1.151, 1.304], < .001	1.213 [1.138, 1.293], < .001
FEV1 (L)	0.306 [0.180, 0.519], < .001	
FEV/FVC	1.052 [0.036, 31.141], 0.977	
Current asthma (Yes vs. No)	1.513 [0.793, 2.885], 0.209	

*BMI* body mass index, *CRP* C-reactive protein, *DBP* diastolic blood pressure, *FEV* forced expiratory volume, *FEV1* forced expiratory volume in 1 s, *FVC* forced vital capacity, *HbA1c* glycated hemoglobin, *HTN* hypertension, *SBP* systolic blood pressure

**Fig. 3:**
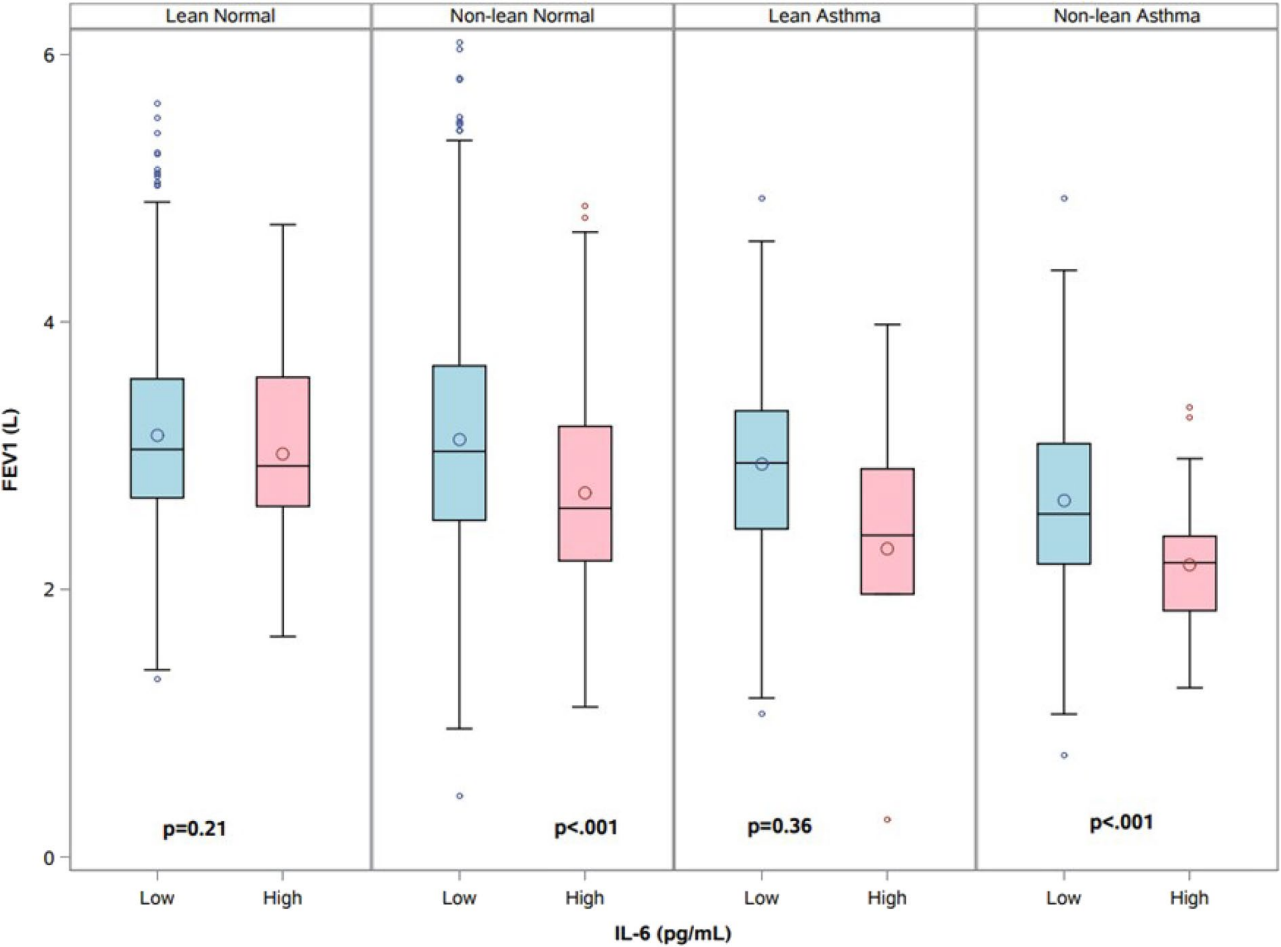
Comparison of lung function between IL-6 (low or high) within asthma and by BMI; *FEV1* forced expiratory volume in 1 s, *IL-6* interleukin 6

To ascertain factors associated with high IL-6 in asthma, we performed logistic regression of high IL-6 patients with asthma only (*n* = 305 or 9% of the cohort) (Table 3). In the univariable models, female sex, Black race, BMI, and CRP were positively associated with the odds of high IL-6 asthma, while FEV_1_ was negatively associated with the odds of high IL-6 (Table 3). However, in the multivariable model, only Black race and CRP were positively correlated with the probability of high IL-6 asthma. We compared lung function between IL-6 levels (high: > 4.979 pg/ml and low: < 4.979 pg/ml) within asthma (presence or absence aka normal) and BMI (lean < 25 kg/m^2^ or non-lean > 25 kg/m^2^) groups (Fig. 3). High IL-6 non-lean patients with asthma had a statistically lower lung function (both FEV_1_ and FVC) compared to low IL-6 non-lean patients with asthma (*p* < 0.001). Even within the lean patients with asthma, having high IL-6 appeared to result in lower FEV_1_; however, this difference was not statistically significant (*p* = 0.3582). Interestingly, we also found that, among the non-lean normal, there was a significantly lower lung function for patients with high IL-6 compared to those with low IL-6 (*p* < 0.001). Finally, multivariable modeling using race-specific cut-off (Black and white) was conducted for high IL-6. (Supplement, Table 4a and b). These models suggested association between the odds of high IL-6 and cRP (*p* < 0.00001, OR 1.103 95% CI 1.064–1.142) for the Black subpopulation and odds of high IL-6 and BMI (*p* < 0.0016, OR 1.057, 95% CI 1.021–1.093), cRP (*p* < 0.0001, OR 1.211 95% CI 1.149–1.276), and FEV1 (*p* < 0.001, OR 0.565, 95% CI 0.406–0.786) for the white subpopulation.

## Discussion

We found significant association between high IL-6 levels and markers of metabolic dysfunction-elevated HbA1c, as well as markers of inflammation, i.e., CRP. This shows the complex interplay between chronic inflammation and metabolic dysregulation in asthma pathogenesis. This also suggests that addressing both inflammatory and metabolic components may be crucial for the optimal management of asthma. Prior cross-sectional studies demonstrated possible connections between obesity, IL-6, and asthma severity [13]. Our study confirmed a positive association between IL-6 and obesity, and further showed significant associations between high IL-6 and features of metabolic syndrome like high blood pressure [18]. Remarkably, the median serum CRP was four times higher in patients with high IL-6. Chronic low-grade inflammation (related to high leptin and low adiponectin) and hormonal dysregulation are possible explanations for this finding [19–21].

The impact of weight on the interaction between IL-6 and asthma is also an important consideration as leptin can increase IL-6 levels [22]. Both lung IL-6 and circulating IL-6 levels are up-regulated in patients with asthma [22]. These observations suggest that IL-6 may play a key role in disease pathogenesis in asthma and obesity-associated asthma (23, 24) Interestingly, established components of metabolic syndrome, like impaired glucose tolerance, increased fasting serum triglycerides, and low high-density lipoprotein levels, are also associated with increased plasma IL-6, raising speculative concerns of increased cardiovascular risk in asthma [24, 25].

IL-6 signaling promotes Th2 cells in select contexts, increases differentiation of effector T cells into pro-inflammatory Th17 subsets, promotes the differentiation of T follicular helper cells, and inhibits generation of regulatory T cells (Treg)[26–28]. IL-6 is thought to evoke immune responses typical of both T2 and non-T2 inflammation as evidenced by increased serum eosinophilia and fraction of exhaled nitric oxide, as well as Th17-mediated neutrophilia in patients with high IL-6 and asthma [29, 30]. IL-6 trans-signaling has been shown to result in greater exacerbation rates and increased expression of genes associated with airway remodeling and obstruction [10]. Interestingly, in a subset of patients with high IL-6, significant T2 inflammation was not identified in the airway epithelium, suggesting a possible link between IL-6 trans-signaling (through sIL-6R) and non-T2 asthma [10, 31]. Neutrophils are purported to be a key component of non-T2 asthma [32] and might be an important source of sIL-6R in the lungs of patients with asthma [33, 34]. High IL-6 was strongly associated with Black race in our analysis. This positive relationship between high IL-6 and race has been previously reported [14], with a higher level of serum IL-6 levels in patients of African American descent. The higher prevalence of obesity and metabolic syndrome in Black individuals, as well as socioeconomic determinants of health, may in part account for the strong association [14, 15].

Several factors were negatively associated with lung function (FEV_1_ and FVC) in our multivariate model, including Black race, CRP, HbA1c, and metabolic dysfunction. The method by which metabolic syndrome impacts lung function could be related to increases in pro-inflammatory cytokines, with contributions from elevated insulin, dyslipidemia, and leptin [35, 36]. Furthermore, studies have shown that a greater number of features of metabolic syndrome are strongly associated with decreased FVC and FEV_1_ in patients with and without chronic pulmonary conditions like COPD and asthma [37]. Moreover, a recent study highlighted the importance of assessing body composition in understanding asthma severity. This study found that severe asthma is associated with higher visceral and subcutaneous fat areas, underscoring the potential role of morph-omics in predicting asthma outcomes [38].

Monoclonal antibodies (mABs) have revolutionized our ability to manage difficult to treat asthma [39]. Those mABs which target IL-6 and its receptor, like sarilumab and tocilizumab, are already used in rheumatological and inflammatory conditions with efficacy [40]. In a small study with two pediatric patients with severe, steroid-resistant asthma, IL-6 blockade with tocilizumab resulted in immunological and clinical improvement of asthma control and exacerbations [41]. Notably, both patients were found to have an IL-4 receptor alpha chain variant, R576 (IL-4Ra-R576), known to drive mixed TH2/TH17 airway inflammation [41, 42]. These observations suggest the plausibility of treating patients with high-IL-6 asthma with mABs, especially given the lack of available options targeting non-T2 asthma. In addition to anti IL-6 mABs, high IL-6 asthma with concomitant obesity and metabolic dysfunction may see further benefits with weight loss and management of metabolic diseases.

### Strengths and Limitations

In the CARDIA cohort, chronic conditions were self-reported including asthma and diseases that constitute metabolic syndrome including hypertension and hypercholesterolemia. Therefore, the cohort was not well characterized using standard definitions or by physician diagnosis or outcomes beyond surrogates such as FEV_1_. Despite this key limitation, we were able to validate prior studies, linking IL-6 to metabolic dysfunction, obesity, and more severe asthma. Furthermore, we were able to demonstrate that IL-6 appears to be higher in the Black population. Compared to prior studies, our cohort included a larger population of healthy individuals from whom the IL-6 cut-off was calculated. Our cohort was also more representative of the Black population, with 46% of patients reporting African American ancestry. However, other races were not represented. Given that there were limited data collected on asthma symptoms and lung function was reported in liters versus percent predicted (which would have adjusted for a patient’s race, height, and age), we were limited in the ability to analyze the data by levels of asthma severity. Data collected on asthma medications were incomplete; more complete data would have afforded an additional method of possibly classifying asthma severity.

## Conclusions

This study underscores the significant association between elevated IL-6 levels and asthma, particularly in the context of obesity and metabolic dysfunction. Our findings indicate that higher IL-6 levels correlate with more severe asthma as assessed by lung function, especially among individuals with obesity. These findings validate a need to better understand the pathologic role of IL-6 in asthma and perhaps the need to identify targeted therapies as being carried out in the PrecISE study (NCT04129931) [43]. Additionally, comprehensive asthma management should address both inflammatory and metabolic factors to improve patient outcomes. Future research should focus on the high IL-6 asthma population that is non-obese and free from metabolic dysfunction to fully understand the additional biological mechanisms at play.

## Supplementary Information

Below is the link to the electronic supplementary material.

Supplementary file1 (PDF 429 KB)

## Data Availability

The datasets generated during and/or analyzed during the current study are available in the BioLinCC repository, [https://biolincc.nhlbi.nih.gov/home/]. This data is not identifiable.
