# Supplementary material for: High Interleukin (IL)-6 is Associated with Lower Lung Function and Increased Likelihood of Metabolic Dysfunction in Asthma
Source: Pulm Ther. 2024 Dec 23;11(1):41–54. doi: 10.1007/s41030-024-00281-z (PMC11861817; doi:10.1007/s41030-024-00281-z)
Supplement: Supplementary file 1 — Supplementary file1 (PDF 429 KB) [file 41030_2024_281_MOESM1_ESM.pdf]

High Interleukin (IL)-6 Is Associated with Lower Lung Function and Increased Likelihood of Metabolic Dysfunction in Asthma

Dionne Adair MBBS [1], AmirBehzad Bagheri MD [2], Matheos Yosef PhD [3], Shokoufeh Khalatbari MS [3], Toby Lewis MD [1], Arjun Mohan MD [2], Njira Lugogo MD [2]

1. Division of Pediatric Pulmonary, Medical College of Georgia, Augusta, GA, USA
2. Division of Pulmonary and Critical Care Medicine, University of Michigan, Ann Arbor, MI, USA
3. Michigan Institute for Clinical and Health Research, University of Michigan, Ann Arbor, MI, USA
4. Division of Pediatric Pulmonology, University of Michigan, Ann Arbor, MI, USA

Corresponding author

Arjun Mohan, MD

Associate Professor, Pulmonary & Critical Care Medicine

University of Michigan

300 North Ingalls St., Suite 2D21, Ann Arbor, MI 48109-5413

Telephone: 734-998-6336

Email: armohan@med.umich.edu

Supplement

Table S1: List of Chronic Conditions Excluded to Define a "Healthy" Subset of The Cohort

**List of Chronic Conditions excluded to define the Healthy population**
ANGINA
ASTHMA
BLOOD CLOT IN LEG VEIN OR LUNG
CANCER (including BLOOD OR LYMPH, BONE, BRAIN, BREAST, CERVICAL, COLON, LUNG, PROSTATE, SKIN, STOMACH, TESTICULAR, UTERINE)
CHRONIC BRONCHITIS
LIVER DISEASE INCLUDING CIRRHOSIS
COPD
DIABETES
DIGESTIVE DISEASE
EMPHYSEMA
GOUT
HIGH CHOLESTEROL
HEART PROBLEM
HEPATITIS
HIV
HYPOTHYROIDISM
HEART ATTACK
HYPERTHYROIDISM
KIDNEY PROBLEM
MELANOMA
MULTIPLE SCLEROSIS
NEPHRITIS OR GLOMERULONEPHRITIS
POLYCYSTIC OVARIAN DISEASE/SYNDROME
PERIPHERAL VASCULAR DISEASE
RHEUMATIC HEART DISEASE
STROKE OR TIA
GASTRIC ULCER

Table S2: Summary of univariable and final multivariable regression models of log(IL-6)

	Univariable (n=3369)			Multivariable (n=3265)	
**Effect**	**N Used**	**Beta [95% CI]**	**P Value**	**Beta [95% CI]**	**P value**
Age at year 20 (years)	3369	0.0026 [-0.0047, 0.0098]	0.484		
Sex (Female vs Male)	3369	0.1322 [0.0803, 0.1841]	<.001		
Race (non-Hispanic Black vs non-Hispanic White)	3369	0.3475 [0.2970, 0.3980]	<.001	0.1148 [0.0641, 0.1655]	<.001
BMI (kg/m2)	3369	0.0497 [0.0461, 0.0533]	<.001	0.0292 [0.0249, 0.0336]	<.001
Asthma (Yes vs No)	3369	0.1240 [0.0472, 0.2007]	0.002		
Asthma in past year (Yes vs No)	3352	0.2289 [0.1274, 0.3305]	<.001		
Currently taking meds for Asthma (Yes vs No)	3360	0.2321 [0.1298, 0.3345]	<.001		
2 or more wheezing attacks (Yes vs No)	3329	0.1608 [0.0747, 0.2469]	<.001		
HTN * (SBP≥130 and DBP≥80)	3366	0.2237 [0.1495, 0.2979]	<.001		
HbA1C (%)	2961	0.1461 [0.1157, 0.1766]	<.001		
Fasting Glucose (mg/dL)	3365	0.0028 [0.0018, 0.0038]	<.001		
Total Plasma Cholesterol (mg/dL)	3367	-0.0008 [-0.0015, -0.0001]	0.033	-0.0010 [-0.0017, -0.0004]	0.002
Average SBP (mm HG)	3366	0.0067 [0.0050, 0.0083]	<.001		
Average DBP (mm HG)	3366	0.0143 [0.0122, 0.0165]	<.001		
Metabolic dysfunction* (Y/N)	3369	0.4276 [0.3645, 0.4907]	<.001	0.1125 [0.0491, 0.1759]	<.001
CRP (ug/mL)	3365	0.0735 [0.0680, 0.0790]	<.001	0.0458 [0.0398, 0.0517]	<.001

	Univariable (n=3369)			Multivariable (n=3265)	
**Effect**	**N Used**	**Beta [95% CI]**	**P Value**	**Beta [95% CI]**	**P value**
FEV1 (L)	3271	-0.2342 [-0.2666, -0.2018]	<.001	-0.0826 [-0.1147, -0.0505]	<.001
FVC (L)	3271	-0.1858 [-0.2106, -0.1611]	<.001		
FEV/FVC	3271	0.5261 [0.1340, 0.9182]	0.009		
Current Asthma (Yes vs No)	3364	0.2301 [0.1378, 0.3223]	<.001		

BMI body mass index, *HTN* hypertension, *HbA1c* glycated hemoglobin, *SBP* systolic blood pressure, *DBP* diastolic blood pressure, *CRP* C-reactive protein, *FEV* forced expiratory volume, *FEV1* forced expiratory volume in 1 second, *FVC* forced vital capacity

Table S3a: Univariable and multivariable regression models of FEV1

**Effect**	**Univariable**				**Multivariable**							
					**Full model**				**Backward elimination (SBC)selected model**			
					**(n=2831)**				**(n=2838)**			
	**Estimate**	**LCL**	**UCL**	**p**	**Estimate**	**LCL**	**UCL**	**p**	**Estimate**	**LCL**	**UCL**	**p**
**Race (non-Hispanic Black vs non-Hispanic White)**	-0.63	-0.68	-0.581	<.0001	-0.492	-0.53	-0.454	<.0001	-0.502	-0.538	-0.467	<.0001
**Sex (Female vs Male)**	-1.004	-1.046	-0.962	<.0001	-0.433	-0.483	-0.384	<.0001	-0.431	-0.479	-0.382	<.0001
**CRP (ug/mL)**	-0.049	-0.055	-0.043	<.0001	-0.009	-0.013	-0.004	0.0002	-0.008	-0.013	-0.004	0.0002

Effect	Univariable				Multivariable							
					Full model (n=2831)				Backward elimination (SBC)selected model (n=2838)			
	Estimate	LCL	UCL	p	Estimate	LCL	UCL	p	Estimate	LCL	UCL	p
**IL-6 (pg/mL)**	-0.091	-0.104	-0.077	<.0001	-0.016	-0.026	-0.007	0.0010	-0.016	-0.026	-0.006	0.0012
**BMI (kg/m2)**	-0.026	-0.03	-0.022	<.0001	0.0116	-0.01	0.0327	0.2839	.	.	.	.
**SUBJECT'S HEIGHT STANDING (CM)**	0.0587	0.0566	0.0608	<.0001	0.0422	0.0345	0.05	<.0001	0.0392	0.0366	0.0418	<.0001
**SUBJECT'S WEIGHT (LBS)**	0.0021	0.0015	0.0027	<.0001	-0.001	-0.005	0.0019	0.4009	.	.	.	.
**HbA1C (%)**	-0.116	-0.147	-0.084	<.0001	-0.053	-0.086	-0.021	0.0015	-0.041	-0.061	-0.021	<.0001
**Fasting Glucose (mg/dL)**	-0.001	-0.002	-12E-6	0.0475	0.0005	-65E-5	0.0016	0.4105	.	.	.	.
**Asthma (Yes vs No)**	-0.416	-0.496	-0.336	<.0001	-0.059	-0.137	0.0195	0.1414	.	.	.	.
**2 or more wheezing attacks (Yes vs No)**	-0.346	-0.436	-0.256	<.0001	-0.111	-0.185	-0.036	0.0036	-0.121	-0.189	-0.053	0.0005
**Average SBP (mm HG)**	-0.003	-0.004	-77E-5	0.0049	0.0005	-0.002	0.0027	0.6809	.	.	.	.
**Average DBP (mm HG)**	-0.01	-0.012	-0.007	<.0001	-0.003	-0.006	-25E-5	0.0329	.	.	.	.
**Metabolic dysfunction* (Y/N)**	-0.137	-0.205	-0.07	<.0001	-0.123	-0.175	-0.071	<.0001	-0.129	-0.175	-0.084	<.0001
**HTN * (SBP≥130 and DBP≥80)**	-0.238	-0.316	-0.16	<.0001	-0.003	-0.074	0.0682	0.9410	.	.	.	.

Effect	Univariable				Multivariable							
					Full model				Backward elimination (SBC)selected model			
					(n=2831)				(n=2838)			
	Estimate	LCL	UCL	p	Estimate	LCL	UCL	p	Estimate	LCL	UCL	p
**Age at year 20 (years)**	-0.014	-0.021	-0.006	0.0004	-0.025	-0.03	-0.02	<.0001	-0.025	-0.03	-0.02	<.0001
**Total Plasma Cholesterol (mg/dL)**	-23E-5	-0.001	0.0005	0.5569	0.0001	-37E-5	0.0006	0.6240	.	.	.	.
**Current Asthma (Yes vs No)**	-0.506	-0.603	-0.409	<.0001	-0.133	-0.223	-0.044	0.0034	-0.178	-0.253	-0.102	<.0001
**Intercept**	.	.	.	.	-2.098	-3.452	-0.744	0.0024	-1.677	-2.207	-1.147	<.0001

Note: in a similar selection model, Average Diastolic Blood Pressure also made it with p=0.0038.

BMI body mass index, *HTN* hypertension, *HbA1c* glycated hemoglobin, *SBP* systolic blood pressure, *DBP* diastolic blood pressure

Table 3b: Univariable and multivariable regression models of FVC

Effect	Univariable				Multivariable							
					Full model				Backward elimination (SL=.05)selected			
	.				(n=2831)				(n=2862)			
	Estimate	LCL	UCL	p	Estimate	LCL	UCL	p	Estimate	LCL	UCL	p
**Race (non-Hispanic Black vs non-Hispanic White)**	-0.886	-0.95	-0.822	<.0001	-0.684	-0.729	-0.638	<.0001	-0.686	-0.73	-0.643	<.0001

Effect	Univariable				Multivariable							
					Full model				Backward elimination (SL=.05)selected			
	.				(n=2831)				(n=2862)			
	Estimate	LCL	UCL	p	Estimate	LCL	UCL	p	Estimate	LCL	UCL	p
**Sex (Female vs Male)**	-1.356	-1.41	-1.303	<.0001	-0.582	-0.641	-0.523	<.0001	-0.589	-0.647	-0.531	<.0001
**CRP (ug/mL)**	-0.067	-0.075	-0.059	<.0001	-0.011	-0.017	-0.006	<.0001	-0.012	-0.017	-0.007	<.0001
**IL-6 (pg/mL)**	-0.122	-0.139	-0.104	<.0001	-0.016	-0.028	-0.004	0.0073	-0.017	-0.028	-0.005	0.0043
**Log(IL-6)**	-0.334	-0.379	-0.29	<.0001	.	.	.	.	.	.	.	.
**BMI (kg/m2)**	-0.04	-0.045	-0.035	<.0001	0.0216	-0.004	0.0469	0.0932	.	.	.	.
**SUBJECT'S HEIGHT STANDING (CM)**	0.0801	0.0775	0.0828	<.0001	0.0629	0.0536	0.0721	<.0001	0.0542	0.0511	0.0573	<.0001
**SUBJECT'S WEIGHT (LBS)**	0.0022	0.0014	0.003	<.0001	-0.004	-0.008	314E-7	0.0518	.	.	.	.
**HbA1C (%)**	-0.169	-0.21	-0.127	<.0001	-0.059	-0.098	-0.02	0.0032	-0.062	-0.086	-0.038	<.0001
**Fasting Glucose (mg/dL)**	-0.002	-0.003	-59E-5	0.0047	-69E-6	-0.001	0.0013	0.9194	.	.	.	.
**Asthma (Yes vs No)**	-0.337	-0.442	-0.231	<.0001	0.0405	-0.053	0.1339	0.3948	.	.	.	.
**2 or more wheezing attacks (Yes vs No)**	-0.24	-0.358	-0.121	<.0001	-0.048	-0.137	0.0413	0.2935	.	.	.	.
**Average SBP (mm HG)**	-0.003	-0.006	-0.001	0.0042	0.0014	-0.001	0.0041	0.3069	.	.	.	.

Effect	Univariable				Multivariable							
					Full model				Backward elimination (SL=.05)selected			
	.				(n=2831)				(n=2862)			
	Estimate	LCL	UCL	p	Estimate	LCL	UCL	p	Estimate	LCL	UCL	p
**Average DBP (mm HG)**	-0.013	-0.016	-0.01	<.0001	-0.003	-0.007	541E-7	0.0538	-0.003	-0.005	-0.001	0.0023
**Metabolic dysfunction* (Y/N)**	-0.234	-0.322	-0.146	<.0001	-0.179	-0.241	-0.117	<.0001	-0.204	-0.261	-0.147	<.0001
**HTN * (SBP≥130 and DBP≥80)**	-0.345	-0.446	-0.243	<.0001	-0.052	-0.137	0.0323	0.2259	.	.	.	.
**Age at year 20 (years)**	-0.007	-0.017	0.0027	0.1548	-0.023	-0.029	-0.017	<.0001	-0.022	-0.028	-0.017	<.0001
**Total Plasma Cholesterol (mg/dL)**	-33E-5	-0.001	0.0007	0.5197	0.0001	-45E-5	0.0007	0.6550	.	.	.	.
**Current Asthma (Yes vs No)**	-0.447	-0.575	-0.319	<.0001	-0.112	-0.219	-0.006	0.0392	-0.112	-0.187	-0.037	0.0035
**Intercept**	.	.	.	.	-4.524	-6.14	-2.907	<.0001	-2.998	-3.641	-2.354	<.0001

CRP C-reactive protein, *BMI* body mass index, *HbA1c* glycated hemoglobin, *SBP* systolic blood pressure, *DBP* diastolic blood pressure, *HTN* hypertension, *LCL* lower control limit, *UCL* upper control limit

Table 4a: Multivariable logistic models of High IL6 for Black individuals

	model			
	1 (Global)<br>(n=1227 out of 1544)		2 (Stepwise)<br>(n=1541 out of 1544)	
**Variable**	**OR [95% CI]**	**p value**	**OR [95% CI]**	**p value**
Age at year 20 (years)	1.041 [0.969, 1.118]	0.2680		

	model			
	1 (Global)<br>(n=1227 out of 1544)		2 (Stepwise)<br>(n=1541 out of 1544)	
Variable	OR [95% CI]	p value	OR [95% CI]	p value
Sex (Female vs Male)	0.686 [0.327, 1.439]	0.3184		
BMI (kg/m2)	1.005 [0.963, 1.048]	0.8174		
Asthma (Yes vs No)	0.579 [0.182, 1.845]	0.3558		
2 or more wheezing attacks (Yes vs No)	1.490 [0.545, 4.071]	0.4366		
HTN * (SBP≥130 and DBP≥80)	0.992 [0.357, 2.751]	0.9871		
HbA1C (%)	1.075 [0.700, 1.653]	0.7407		
Fasting Glucose (mg/dL)	0.995 [0.979, 1.010]	0.4888		
Total Plasma Cholesterol (mg/dL)	0.993 [0.986, 1.000]	0.0673		
Average SBP (mm HG)	0.993 [0.961, 1.025]	0.6542		
Average DBP (mm HG)	0.998 [0.957, 1.042]	0.9355		
Metabolic dysfunction* (Y/N)	0.946 [0.454, 1.972]	0.8819		
cRP (ug/mL)	1.103 [1.064, 1.142]	<.0001	1.114 [1.083, 1.147]	<.0001
FEV1 (L)	0.943 [0.553, 1.607]	0.8293		
FEV/FVC	1.010 [0.017, 59.365]	0.9961		
Current Asthma (Yes vs No)	1.781 [0.562, 5.648]	0.3268		

Table 4b: Multivariable logistic models of High IL6 for white individuals

	model			
	1 (Global)<br>(n=1604 out of 1825)		2 (Stepwise)<br>(n=1783 out of 1825)	
Variable	OR [95% CI]	p value	OR [95% CI]	p value
Age at year 20 (years)	0.993 [0.946, 1.043]	0.7730		
Sex (Female vs Male)	0.655 [0.406, 1.058]	0.0835	0.614 [0.408, 0.925]	0.0196
BMI (kg/m2)	1.057 [1.021, 1.093]	0.0016	1.066 [1.038, 1.095]	<.0001
Asthma (Yes vs No)	0.605 [0.263, 1.391]	0.2369		
2 or more wheezing attacks (Yes vs No)	0.805 [0.392, 1.651]	0.5542		
HTN * (SBP≥130 and DBP≥80)	0.454 [0.212, 0.972]	0.0422		
HbA1C (%)	1.427 [1.040, 1.957]	0.0274		
Fasting Glucose (mg/dL)	0.992 [0.980, 1.003]	0.1635		
Total Plasma Cholesterol (mg/dL)	0.995 [0.990, 1.000]	0.0484		

	model			
	1 (Global) (n=1604 out of 1825)		2 (Stepwise) (n=1783 out of 1825)	
**Variable**	**OR [95% CI]**	**p value**	**OR [95% CI]**	**p value**
Average SBP (mm HG)	1.001 [0.978, 1.024]	0.9592		
Average DBP (mm HG)	1.007 [0.980, 1.034]	0.6302		
Metabolic dysfunction* (Y/N)	1.246 [0.799, 1.942]	0.3324		
cRP (ug/mL)	1.211 [1.149, 1.276]	<.0001	1.201 [1.144, 1.260]	<.0001
FEV1 (L)	0.565 [0.406, 0.786]	0.0007	0.574 [0.431, 0.766]	0.0002
FEV/FVC	0.666 [0.047, 9.409]	0.7639		
Current Asthma (Yes vs No)	1.547 [0.616, 3.884]	0.3527		
